# The Evolving Landscape of Cardiovascular Disease Prevention

**DOI:** 10.14797/mdcvj.383

**Published:** 2021-09-24

**Authors:** Miguel Cainzos-Achirica, Kershaw V. Patel, Khurram Nasir

**Affiliations:** 1 Division of Cardiovascular Prevention and Wellness, Department of Cardiology, Houston Methodist DeBakey Heart & Vascular Center, Houston, TX, US; 2 Center for Outcomes Research, Houston Methodist, Houston, TX, US; 3 Johns Hopkins Ciccarone Center for the Prevention of Cardiovascular Disease, Johns Hopkins Medical Institutions, Baltimore, MD, US

In 2021, cardiovascular disease (CVD) remains the number one cause of death in the United States (US).^1^ Despite nation-wide decreasing trends in cardiovascular mortality since the 1970s, this has recently plateaued, and some projections suggest that there may be an increase in CVD mortality in the coming years.^2^ A significant contributor to this shift is the concerning, rapid rise in the burden of cardiometabolic risk factors such as obesity and diabetes, both of which disproportionately affect racial and ethnic minorities.^3,4^ As the prevalence of diabetes has increased, there has also been a recent rise in rates of acute myocardial infarction (MI) among young and middle-aged adults with diabetes.^5^ Younger adults now account for a larger proportion of US patients hospitalized with a first MI, and CVD represents a major cause of death among women.^6,7^

Given these trends, there is increasing recognition of the critical role of preventive cardiology, and this issue of the *Methodist DeBakey Cardiovascular Journal* focuses on several recent developments in this space. The eight state-of-the-art reviews led by an outstanding panel of authors cover a wide range of topics, from current needs in preventive cardiology training and novel insights on the contextual factors that contribute to the development and prognosis of CVD to most recent updates on lipid-lowering therapy and aspirin, and from cardiovascular prevention in young adults and women in 2021 to the lessons learned during the ongoing Coronavirus Disease 2019 (COVID-19) pandemic and innovative approaches to enhance the management of individuals who have already had a CVD event.

The need for a greater emphasis on the primordial and primary prevention of CVD is impeccably outlined in the first article of this issue, led by Drs. Tahir Mahmood and Michael Shapiro. The authors walk us through key milestones in cardiovascular epidemiology and preventive cardiology, from the landmark Framingham Heart Study to statin trials. They then discuss more recent developments, including advances in atherosclerotic CVD (ASCVD) risk assessment such as subclinical atherosclerosis imaging, novel therapeutics (eg, proprotein convertase subtilisin/kexin type 9 [PCSK9] inhibitors), and exciting research venues moving forward, such as novel genetic editing CRISPR-based techniques. Given a rapidly increasing therapeutic arsenal and the need for a multidisciplinary, highly specialized set of skills to ensure the highest quality preventive cardiology care, the authors make a compelling case about the need for a dedicated, officially recognized subspecialty in preventive cardiology. This is already being implemented in a number of programs across the country^8^; however, fragmentation and lack of standardization are noted as significant issues. The authors also discuss novel notions including the need to expand the focus of cardiovascular prevention from ASCVD to also address other highly prevalent conditions, such as heart failure and atrial fibrillation, and the potential benefits of research networks involving preventive cardiology centers across the country. We applaud these propositions and believe the time has come to implement them.

One of the skills that the modern preventive cardiologist will need to master is a good understanding of the complex social factors that shape the burden and prognosis of ASCVD in patients. There is increasing recognition of the importance of social determinants of health (SDOH) in cardiovascular medicine and specifically in preventive cardiology, and in the second article of this issue, Drs. Rahul Singh, Zulqarnain Javed, and colleagues shed light on the specific role of community and social context, an integral component of most SDOH frameworks.^9^ The authors discuss the protective effects of social support, social cohesion, lack of discrimination, and community engagement in cardiovascular health and outline the potential pathways involved. These mechanisms shape both our susceptibility/resilience to illness and our resources to deal with disease once developed, and the authors stress the need for greater awareness of their importance both at the population and individual-patient levels. Furthermore, the authors discuss policy recommendations aimed at enhancing social support and preventing discrimination, particularly against racial/ethnic minorities, and propose a number of research initiatives aimed at further expanding our understanding of this contextual factor. Beyond groundbreaking therapies and novel biomarkers, a greater attention to the context in which our patients live and care for their health will help further improve prevention and outcomes, especially among groups disproportionately affected by ASCVD.

In addition to social context, genetic factors are also important determinants of the risk of developing ASCVD. This is particularly true among individuals with familial hypercholesterolemia (FH), a monogenic condition that causes very high circulating levels of low-density lipoprotein cholesterol (LDL-C) from birth and is associated with a marked increase in the lifetime risk of ASCVD, including premature presentations.^10^ In their state-of-the-art review titled the “Past, Present, and Future of Familial Hypercholesterolemia Management,” Drs. Viviane Rocha and Raul Santos describe the genetics, epidemiology, pathophysiology, and management of FH, from statins (which in 2021 still represent the cornerstone of therapy in these patients) to more novel drugs—such as PCSK9 and angiopoietin-like protein 3 (ANGPTL3) inhibitors—that can yield dramatic reductions in LDL-C levels in patients in whom statins fall short. Recent studies have revealed that risk is far from homogeneous among individuals with FH,^11,12^ and in the context of costly novel LDL-C–lowering therapies and finite healthcare resources, the authors discuss the potential role that clinical scores, coronary imaging, and blood biomarkers may have for a more personalized and potentially cost-effective allocation of novel LDL-C-lowering drugs in these patients.

Besides lipid-lowering agents, aspirin has been another highly utilized pharmacological intervention in the primary and secondary prevention of ASCVD. Nevertheless, recent research, including three landmark trials published in 2018 (ASPREE, ASCEND, and ARRIVE), suggests that in the current era of widespread statin use, enhanced management of risk factors (such as hypertension and diabetes), anti-tobacco laws, and decreasing ASCVD event rates in most Western populations, aspirin’s relevance is shrinking in primary prevention.^13^ In their elegant, richly illustrated article, Drs. Ella Murphy, James Curneen, and John McEvoy discuss these trends and contrast them with recent analyses in the Multi-Ethnic Study of Atherosclerosis and Dallas Heart Study, which suggest that a careful selection of low-bleeding–risk candidates together with the prognostic information provided by coronary artery calcium (CAC) score can help implement the American College of Cardiology/American Heart Association (ACC/AHA) class IIb recommendation to consider low-dose aspirin among primary prevention individuals at highest risk of ASCVD events who are not at high risk of bleeding.^14,15,16^ In secondary prevention settings, the evidence is more robust and aspirin is a more established therapy. However, with recent developments in antithrombotic therapies such as P2Y_12_ inhibitors and declining rates of recurrent ASCVD events, the authors discuss how the recommendation for chronic therapy with aspirin after a first acute/chronic coronary syndrome may need to be re-evaluated in the near future.

Another increasingly important area of research and innovation in preventive cardiology in 2021 encompasses the prevention of ASCVD in younger adults. In the US, younger patients now comprise a growing proportion of those hospitalized with a first MI,^6^ and clinical registries such as YOUNG-MI suggest that current risk estimation and management algorithms, such as the Pooled Cohort Equations and the associated ACC/AHA treatment thresholds, fail to consider many high-risk young adults as good candidates for preventive statin therapy before they have had a first MI.^17,18^ Consequently, there is increasing interest in characterizing the risk profile of men and women who develop MIs at early ages as well as in enhancing our societal ability to prevent premature events.^19^ To address this, Theresa Rizk and Dr. Ron Blankstein present us with an expert overview of this topic, which includes a systematic review of the latest literature in this field. As noted by the authors, a particularly relevant finding from a prevention standpoint is the finding, reported in multiple studies, of most patients with premature MIs having untreated traditional risk factors, especially tobacco use.^20,21^ This suggests that population-level interventions aimed at reducing first- and second-hand exposure to tobacco products, together with a timely, exhaustive screening and management of other traditional risk factors (eg, dyslipidemia, family history of ASCVD) in young adults can help dramatically curtail the rates of premature ASCVD. In addition, the authors discuss the potential role of the CAC score for further risk assessment in specific young adults, although available data in young individuals without a family history of ASCVD is mostly derived from clinical cohorts rather than from general population-based studies.

Of note, Rizk and Blankstein describe important differences in the risk factor profile and outcomes of men and women who develop premature MIs. Building on this, in their comprehensive women’s health review, Drs. Lochan Shah, Garima Sharma, and colleagues present a thorough update on the current knowledge of cardiovascular risk factors specific to women. On one hand, the authors make a compelling case for the need to screen traditional risk factors before and during pregnancy because those are associated with a variety of adverse pregnancy outcomes including preeclampsia, gestational hypertension/other hypertensive diseases of pregnancy, gestational diabetes mellitus, preterm birth, small-for-gestational-age infant, placental abruption, ischemic stroke, and maternal and neonatal mortality, among other complications. On the other hand, maternal adverse pregnancy outcomes, polycystic ovarian syndrome, and premature menopause represent independent risk factors for CVD later in life,^16^ and they should be screened systematically as part of a standard risk assessment visit for women. The authors provide suggestions for risk mitigation during pregnancy in women with cardiovascular risk factors as well as in nonpregnant patients with women-specific risk factors, and we believe the readership of the *Methodist DeBakey Cardiovascular Journal* will find this guidance extremely useful. Finally, the authors identify the pregnancy and peripartum period as a window of opportunity in which women may be more receptive to lifestyle change and other cardiovascular health recommendations.

In August 2021, the COVID-19 pandemic continues to permeate all aspects of life and medicine and has a direct impact in the way we live, interact with each other, treat patients, and prevent disease.^22^ Drs. Eamon Duffy, Erin Michos, and colleagues discuss how this worldwide health crisis has also negatively disrupted preventive cardiology care, particularly during its initial months. Throughout lockdowns, many patients missed in-person visits, underwent fewer assessments of cardiovascular risk factors, reduced their levels of physical activity, increased their snacking and caloric intake, and felt isolated and stressed at home. Despite these challenges, the authors also describe important lessons learned and opportunities to enhance the prevention of ASCVD moving forward. Indeed, the pandemic has reshaped preventive cardiology, introducing almost overnight long-overdue improvements that can facilitate a more personalized and effective approach moving forward. Telemedicine has become a critical component of the “new normal,” and while it should not fully replace in-person interactions, it provides a powerful platform that can facilitate closer follow-up of higher-risk patients and earlier detection of poorly controlled risk factors and newly developed symptoms. For some, the pandemic was also an opportunity to quit tobacco and increase levels of physical activity at home using stationary bicycles and other devices, although more intensive public health efforts will be needed to expand these positive changes to larger populations and sustain them over time. The authors also discuss opportunities to leverage currently increased health awareness among the general population to enhance ASCVD prevention efforts, and stress the importance of COVID-19 vaccination as a critical step towards preventing morbidity and death — particularly among the elderly, minorities, and those with multiple cardiovascular risk factors and/or with established ASCVD.

Finally, what about developments for secondary ASCVD prevention among individuals who have already had a first event? A wealth of interventions is now available for these patients, and clinical practice guidelines from cardiovascular scientific societies are updated periodically to accommodate the most recent evidence. However, US and international registries reveal that the implementation of guideline recommendations among patients with established ASCVD remains far from optimal in most countries.^23^ To ameliorate this, Drs. Mohamad Taha, Khurram Nasir, and colleagues present an innovative checklist-based approach aimed at facilitating a more systematic and complete implementation of evidence-based interventions. Checklists, which were initially developed in aviation, have been successfully implemented in other fields of medicine such as surgery and intensive care units. Several supporting materials are also proposed, including periodic quality and performance reports that can help identify specific implementation gaps and inform targeted timely improvements. Although formal research is needed to confirm the effectiveness of this strategy, in a context of typically busy preventive cardiology clinics, this approach can be a powerful aid for preventive cardiology professionals, potentially increasing the use of classic and novel interventions in secondary prevention, minimizing therapeutic inertia, and improving patient outcomes.

We would like to thank the editors of the *Methodist DeBakey Cardiovascular Journal* for their vision and support of preventive cardiology endeavors through this focused issue. We also thank the authors of the eight articles for their fabulous work and generous contributions to this issue, which we believe will provide our readers with a comprehensive overview of several landmark developments in this space. We hope this issue will help raise further awareness on critical topics such as SDOH, women’s cardiovascular health, the importance of COVID-19 vaccination, and the need for a more systematic implementation of existing guideline recommendations in primary and secondary prevention. Finally, we hope we were able to *infect* the readership with our enthusiasm moving forward. These are unprecedented times, but also very exciting ones in the field of preventive cardiology.

## Biographies of Editors

The editors of the Methodist DeBakey *Cardiovascular Journal* thank Drs. Khurram Nasir, Miguel Cainzos-Achirica, and Kershaw Patel for their enthusiasm and dedication in curating this issue on cardiovascular disease (CVD) prevention.

**KHURRAM NASIR, MD, MPH, MSC** 

**Figure:**
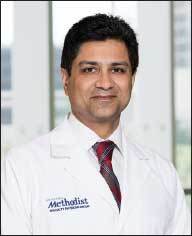


Khurram Nasir, MD, MPH, MSc, holds a broad range of leadership positions at Houston Methodist focusing on population health and preventive cardiology. He is the chief of the Division of Cardiovascular Prevention and Wellness in the Department of Cardiology, division chief of Health Equity and Disparities Research and co-director at the Center for Outcomes Research, and director of the Center for Cardiovascular Computation & Precision Health. He is also a professor of cardiology and Jerold B. Katz Investigator at the Houston Methodist Academic Institute. His research interests cover a range of topics including translation, population health, big data, and social aspects of cardiovascular prevention.

Dr. Nasir earned his medical degree at Allama Iqbal Medical College in Pakistan followed by a Master of Public Health at Johns Hopkins University. He completed his internal medicine residency at Boston Medical Center and cardiology fellowship at Yale University. He then moved on to postdoctoral research training at Johns Hopkins Hospital and received a National Institutes of Health (NIH) T32 fellowship in cardiac imaging at Massachusetts General Hospital. In 2017, he continued his education with a Master of Science in health economics and policy management from the London School of Economics and Political Science.

A prolific researcher, Dr. Nasir has published more than 730 peer-reviewed articles in leading cardiovascular journals and is an associate editor for *Circulation: Quality of Care and Outcomes*; serves on the editorial boards of the *Methodist DeBakey Cardiovascular Journal and Circulation*; and is on the board of directors for the American Society of Preventative Cardiology. In recognition of his effort, he was recognized with the Johns Hopkins Distinguished Alumni Award in 2013, which honors alumni who have typified the Johns Hopkins tradition of excellence and brought credit to the University by their personal accomplishment, professional achievement, or humanitarian services. In 2020, he received the Arthur S. Agatston Cardiovascular Disease Prevention Award that recognizes individuals whose pioneering efforts have saved lives from the leading killer throughout the world, coronary artery disease.

**MIGUEL CAINZOS-ACHIRICA, MD, MPH, PHD** 

**Figure:**
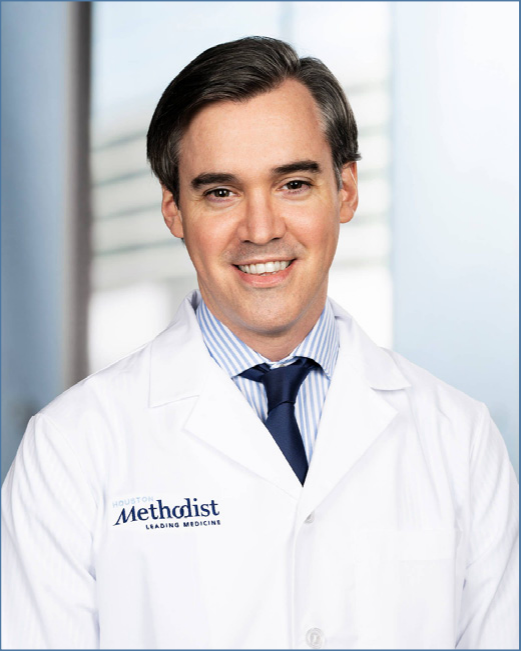


Miguel Cainzos-Achirica, MD, MPH, PhD, joined the Houston Methodist faculty in 2020 after completing his training in cardiology and cardiovascular epidemiology at leading academic institutions in the United States and Europe. He serves as associate director of Preventive Cardiology Research and Education at the Houston Methodist DeBakey Heart & Vascular Center and is an assistant professor of Preventive Cardiology at Cornell Weill and the Houston Methodist Center for Outcomes Research. He is also an adjunct assistant professor of medicine at Johns Hopkins University. With training in cardiology, epidemiology, biostatistics, cardiac imaging, and minority health, Dr. Cainzos-Achirica has research interests in CVD prevention, risk assessment, use of cardiac imaging modalities (particularly computed tomography) to enhance cardiovascular risk stratification, CVD prevention among vulnerable populations (especially people of South Asian ancestry), and advanced epidemiological research methods.

After earning his medical degree from the University of Santiago de Compostela in Spain, Dr. Cainzos-Achirica trained in cardiology in Barcelona, where he also completed a diploma in biostatistics and study design at the Autonomous University of Barcelona. He then moved to the United States, where he completed a Master of Public Health at the Johns Hopkins Bloomberg School of Public Health followed by 2 years of postdoctoral training in preventive cardiology research at the Johns Hopkins School of Medicine and Welch Center for Prevention. He also holds a Doctor of Philosophy in cardiovascular epidemiology from the Universitat de Barcelona and is a prospective student in the prestigious MSc in Clinical Trials program at the University of Oxford. Dr. Cainzos- Achirica has received multiple performance awards from public and private organizations and competitive training grants from the Spanish Society of Cardiology (2014), the Caixabank Foundation (2015), and the European Society of Cardiology (2021).

Dr. Cainzos-Achirica has published more than 130 peer-reviewed articles, including original science, state-of-the-art review papers, invited commentaries, and research methods letters in the highest impact cardiovascular and medical peer-reviewed journals.

**KERSHAW PATEL, MD, MS** 

**Figure:**
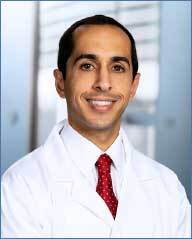


Like Dr. Cainzos-Achirica, Kershaw Patel, MD, MS, joined the preventive cardiology team at Houston Methodist DeBakey Heart & Vascular Center in 2020. He is a cardiologist with Houston Methodist DeBakey Cardiology Associates in the hospital’s Division of Cardiovascular Prevention & Wellness, assistant professor of cardiology at The Houston Methodist Academic Institute, assistant professor of medicine at Weill Cornell Medical College, and an assistant clinical member of the Houston Methodist Research Institute. Dr. Patel’s clinical and research interests are in heart failure prevention in highrisk populations, including people with diabetes.

Dr. Patel earned his medical degree at the University of Texas (UT) Medical Branch at Galveston and fulfilled his residency at the University of Chicago. He went on to complete a cardiovascular disease fellowship at UT Southwestern Medical Center in Dallas, where he also earned a Master of Science in clinical science.

At Houston Methodist, Dr. Patel has been honored with the Academic Institute’s President’s Award for Excellence in Peer-Reviewed Publication and the Clinician Trialist Award. He was also a finalist in the American Heart Association’s (AHA) Lifestyle and Cardiometabolic Health Early Career Investigator Award Competition and won the AHA Get With the Guidelines Young Investigator Research Seed Grant Award.

To date, Dr. Patel has published more than 30 manuscripts, many with original research on CVD and heart failure risk assessment, including focused work on cardiometabolic diseases such as type 2 diabetes and obesity.

We are grateful to these three exceptional guest editors for their leadership and dedication and to the many authors who contributed their time and expertise to creating this comprehensive exploration of the present and future of cardiovascular disease prevention.
